# Findings in patients with chronic intestinal dysmotility investigated by capsule endoscopy

**DOI:** 10.1186/1471-230X-7-29

**Published:** 2007-07-18

**Authors:** Charlotte M Hoog, Greger Lindberg, Urban Sjoqvist

**Affiliations:** 1 Stockholm South Hospital, Karolinska Institutet, Stockholm, Sweden; 2 Karolinska Institutet, Department of Medicine, Karolinska University Hospital Huddinge, Stockholm, Sweden

## Abstract

**Background:**

Capsule endoscopy (CE) is a unique tool to visualize the mucosa of the small intestine. Chronic intestinal dysmotility (CID) is a group of rare disorders of gastrointestinal motility that often are complicated by bacterial overgrowth. The aim of this study was to determine the prevalence of small bowel mucosal abnormalities in patients with CID. We also studied the usefulness of CE in the diagnosis of intestinal dysmotility.

**Methods:**

We conducted a prospective study using CE in 18 patients; six with myopathic, 11 with neuropathic and one with indeterminate CID. A control group was used for comparison of small bowel transit.

**Results:**

Mucosal breaks (erosions and ulcerations) were found in 16/18 (89%) patients. The capsule reached the caecum in 11/18 (61%) patients with a median transit time of 346 minutes. In the control group the capsule reached the caecum in 29/36 (81%) cases with a median transit time of 241 minutes. The difference in transit time was not significant (p = 0.061) in this material. The capsule was retained in the stomach in 3/18 patients. None of the patients developed symptoms or signs of mechanical obstruction.

**Conclusion:**

A high frequency of mucosal breaks and signs of motility disturbances were seen in CID patients. CE is feasible for the examination of small bowel mucosa in patients with CID. The relevance of observed mucosal abnormalities in CID remains uncertain.

## Background

Chronic intestinal dysmotility (CID) is a syndrome that is characterized by symptoms and signs of intestinal obstruction in the absence of a mechanical blockage [1]. CID is caused by abnormalities in the intestinal smooth muscle or the myenteric plexus, usually affecting selectively one of them [2]. The underlying pathology in CID is thus believed to comprise two major types: myopathic and neuropathic disorders, although they usually present with similar clinical manifestations [3]. There is considerable confusion regarding the nomenclature in gastrointestinal motility disorders. Patients with CID can also be divided into those with chronic intestinal pseudo-obstruction (CIP) and those with enteric dysmotility (ED) [4]. At present the possible medical or surgical treatment for this complex and often debilitating syndrome is limited [1,5,6].

Wireless capsule endoscopy (CE) is a method to examine the mucosa of the small intestine. The patient swallows a small capsule containing a video camera that takes two frames per second during its journey through the gastrointestinal tract. It is propelled by peristalsis and disposable. This method, first described by Iddan et al [7], is now widespread and has revolutionized the visualisation of small intestinal mucosa.

Previously CID has been considered a contraindication for CE [8-10]. In the literature there is only one published original article where capsule endoscopy has been performed in patients with CID [11]. In this study six patients were examined and found to have a high frequency of mucosal breaks. The knowledge of the appearance of the mucosa in vivo in this condition is therefore incomplete and the experience of performing CE in patients with CID seems to be limited.

In this prospective study we examined patients with known CID by means of CE. The primary aim of our study was to evaluate the small bowel mucosa of patients with CID. A secondary aim was to find out if CE, by evaluating small bowel transit and signs of dysmotility, could differentiate the two histopathological types of CID from each other and from a control group.

## Methods

Eighteen patients with CID were examined with CE for the purpose of this study. The patients had a well documented motility disorder. Their diagnosis was based on clinical features, x-ray findings, small-bowel manometry and intestinal full thickness biopsy. Six of them had myopathic CID, 11 had neuropathic CID and one had indeterminate CID. Their ages ranged between 35–85 (median 54) years. There were 6 males and 12 females. One female had a previous history of gastro-intestinal bleeding, whereas the others had no such history.

Including intestinal full thickness biopsy the patients had undergone abdominal surgical interventions 1–10 (median 2) times (Table 1). Surgery had been performed for different reasons including cholecystectomy, appendectomy and gynaecological interventions. In five cases (#11, #12, #14, #16, #18) surgery was aimed at treating the underlying motility disorder and consisted mainly of bowel resections.

**Table 1:** Clinical characteristics and results of capsule endoscopy in 18 patients with chronic intestinal dysmotility.

Nr	Gender/Age	Type of CID	Previous Abdom. Surgery	View (1)	Caecum (2)	Transit time (3)	Retention (4)	Ulcers	Erosions	Miscellanous
1	M/39	Myopathy	2	Clear	Yes	378			19	
2	M/59	Myopathy	3	Poor	No				2	Inflam (6)
3	M/46	Myopathy	1	Med	Yes	219	S 165		1	
4	F/41	Myopathy	2	Poor	No				1	
5	F/72	Myopathy	2	Med	No				2	Angiodysplasia
6	M/85	Myopathy	1	Poor	No		O 20		1	
7	F/52	Neuropathy	2	Clear	Yes	305			4	
8	F/59	Neuropathy	2	Clear	Yes	353	S 480		5	CE × 2 (7)
9	M/49	Neuropathy	4	Clear	Yes	363		2		
10	F/56	Neuropathy	2	Clear	Yes	209	S 180		1	
11	F/36	Neuropathy	4 (5)	Clear	Yes	197			2	
12	F/65	Neuropathy	2 (5)	Med	No				10	
13	F/47	Neuropathy	3	Poor	Yes	335				
14	F/35	Neuropathy	6 (5)	Poor	No				3	
15	F/65	Neuropathy	1	Clear	Yes	227			1	Inflam
16	F/57	Neuropathy	10 (5)	Clear	Yes	286				Angiodysplasia Flebectesia
17	F/40	Neuropathy	4	Clear	Yes	338		8	10	
18	M/69	Indeterminate	6 (5)	Clear	No				6	

(1) Clear = Clear view, small amounts of intestinal content. Med = Medium view, some intestinal content hiding parts of the mucosa. Poor = Poor view, large amounts of intestinal content hiding a considerable part of the mucosa.

(2) The capsule reached caecum within recording time.

(3) Transit time in small intestine in minutes.

(4) O = oesophagus, S = stomach, retention measured in minutes.

(5) Surgery included intestinal resections.

(6) Inflam = Inflammation, signs of villus oedema and redness.

(7) First capsule was retained in the stomach.

No drugs that could interfere with motility were allowed for 48 hours before examination. Non steroid anti-inflammatory drugs (NSAIDs) were not allowed but one of the patients (#17) was later found out to have been using this at the time for capsule endoscopy. The patients had only liquid food on the evening before and no oral intake at all from midnight before examination. No bowel preparation was given. The capsule (PillCam, Given Imaging) was swallowed with two glasses of water.

The capsule takes two images per second during eight hours, depending on battery power. The frames are transmitted directly to a recorder and after the examination they are unloaded onto a computer were the approximately 50,000 frames can be viewed one by one or as a video sequence.

The results from the CE examinations were viewed by two of us, first separately and then together. Only findings that both agreed to be significant were recorded as present. The readers were blinded for the type of CID the patients were suffering from as well as all other clinical information.

If the capsule did not reach the caecum during the recording time, complete small intestinal passage was controlled by means of fluoroscopy, which was carried out within a week after the examination.

In order to evaluate small bowel transit in the study cases, a control group was used. The control group consisted of 36, randomly selected, age and gender matched patients who previously had underwent capsule endoscopy at our centre, between 2003 and 2005, because of occult gastrointestinal bleeding. The controls had been viewed by the same two readers as the study group and the original results concerning small bowel transit were used. The patients had no symptoms of intestinal obstruction or history of motility disorder. The CE procedure, including bowel preparation, was the same in both groups.

### Statistical analysis

Median values with ranges were used in the text. We used Kaplan-Meier plots and the log-rank test for the analysis of small bowel transit times.

### Ethical consideration

This study was approved by The Research Ethics Committee of Karolinska Institutet, Stockholm, Sweden. The participants were given oral and written information and have given their consent orally.

## Results

All patients underwent the examination without complications. None of them developed symptoms of intestinal obstruction during the examination and no one needed endoscopical or surgical removal of the capsule. The results are presented in detail in Table 1.

Three patients retained the capsule in the stomach (defined in this study as more than two hours in the stomach), one of them for the whole recording time. That patient underwent a new CE with endoscopic placement of the capsule in the duodenum and the second capsule reached caecum within recording time. One patient had retention of the capsule in the oesophagus for 20 minutes.

The capsule reached the caecum during the eight-hour recording time in 11/18 (61%) patients. In patients with myopathic CID the capsule reached the caecum during the recording time in only 2/6 patients, despite that only one had a gastric retention. In the control group the capsule reached the caecum in 29/36 (81%) patients. When the capsule did not reached the caecum during the recording time, the examination was considered as incomplete and the small bowel transit time was estimated to >400 min. The median transit time in neuropathic CID was 305 (197 – >400) minutes whereas in myopathic CID the median transit time was >400 (219 – >400) minutes (p = 0.051). In the whole study group median transit time was 346 (197 – >400) minutes, whereas in the control group it was 241 (75 – >400) minutes (p = 0.061).

The view was considered as clear with little or no intestinal content in only 10/18 (56%) patients. In cases where the view was reduced the reason was mainly large amounts of intestinal content. In the control group the view was considered as clear in 30/36 (83%) patients.

**Figure 1:**
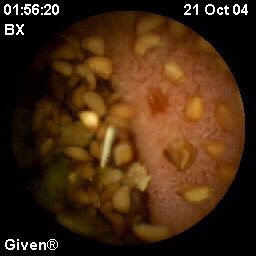
**Erosion**. Erosion in the small intestine, detected by capsule endoscopy in a patient with chronic intestinal dysmotility and large amounts of intestinal content.

**Figure 2:**
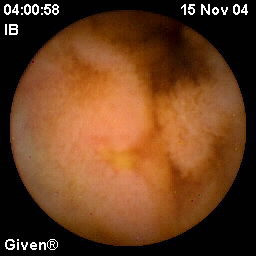
**Ulcer**. Ulcer in the small intestine as seen by capsule endoscopy in a patient with chronic intestinal dysmotility.

Mucosal breaks were found in 16/18 (89%) patients and in seven patients there were multiple lesions (three or more). The mucosal breaks were divided into erosions and ulcerations. The findings defined as erosions included not only redness but also some loss of mucosa (Figure 1) and often could a small fibrin clot be seen. Ulcerations, on the other hand, were larger, deeper and covered with fibrin (Figure 2). Erosions were the dominating type of mucosal breaks that was found. At most, one patient had 19 erosions. In patient #17, eight ulcerations were found. This patient, however, was found to have used NSAIDs during the period before CE examination. The exact size and localization of lesions are not possible to determine by means of CE and subsequently not in this study group either.

Other findings included angiodysplasias and flebectasias. In patient #16, who had a history of occult gastrointestinal bleeding, multiple angiodysplasias were found. Two of the patients showed signs of inflammation with swollen villi and reddish mucosa.

## Discussion

This is a descriptive study in a limited number of patients, but so far the largest systematic study using capsule endoscopy in patients with chronic intestinal dysmotility presented in the literature.

The capsule reached the caecum in 61% of the patients compared to 81% in the control group. Previous studies in mixed patient groups have found passage to the caecum in 75–95% [8,12-19]. In the literature mean transit time is usually measured in studies comparing bowel preparation and the use of pro-kinetic drugs, and varies between 218 and 255 minutes [14-18]. Since mean transit time was not possible to determine in this study, because of the large number of incomplete examinations, where transit time only was known to be 400 minutes or more, we chose to use median transit time instead. A tendency towards prolonged transit time was seen in CID patients compared to controls.

When the capsule does not reach the caecum during the recording time, the diagnostic yield of CE may be decreased [19]. Large amounts of intestinal content may also impair the view and lead to a higher risk for overlooking lesions, thus decreasing the diagnostic yield of CE. Despite these limitations, we found that 89% of the patients had mucosal breaks and this was an unexpected finding. Forty-four percent (8/18) of our patients had three or more erosions or deeper lesions that were classified as ulcers. In a group of 413 healthy volunteers who underwent CE, Goldstein et al [20] found mucosal breaks in only 14%. One of our patients with multiple ulcers was later found out to have been using NSAIDs when performing the examination and this is a well-known cause for ulcers in the small intestine [20-23]. The other patients had no obvious reason for presenting mucosal breaks. The reasons for the large proportion of mucosal breaks in this study are only speculative. One possible explanation is that the mucosal breaks are due to bacterial overgrowth, which is common in CID as a complication of the impaired transit [24].

Comparing the two histopathological groups of CID was difficult because of the limited size of the study group and its dominance of neuropathic cases. A prolonged median transit time was seen in the myopathic group but the difference did not quite reach statistical significance (p = 0.051). Patients with myopathic CID had more often large amount of intestinal content and because of that, did not present a clear view in half of the cases. No differences regarding mucosal breaks could be noticed between the two groups.

Motility disturbances are thus detectable by means of CE although the method was unable to confirm the diagnosis or differentiate between the two forms of CID in this study. CE will probably not be a first line examination tool in diagnosing CID but might be indicated in selective cases.

As mentioned in the introduction CID has been considered as a contraindication for CE. The reason for this should be the supposed high risk for capsule retention. Patients diagnosed with CID have been thoroughly examined without findings of narrowing strictures and per definition they do not have mechanical obstruction. On the other hand traditional x-ray methods like small bowel follow through and enteroclysis have recently been shown to have a low sensitivity for intestinal strictures [13,17,25-28]. If the lumen is narrowed because of a lesion the capsule might be permanently retained (defined as retention > 2 weeks) and surgical or endoscopic removal becomes necessary. Permanent retention is reported to occur in 0 to 13% of all CE procedures, depending on the definition for capsule retention and how the patients were selected [12,13,25-31]. In recent years CE has been performed in a large number of patients with known or suspected Crohn's disease [26,28,29,32] and even in some cases with suspected or known strictures [26,28]. However there are, to our knowledge, only two cases of acute small bowel obstruction requiring acute surgery following a CE procedure published in the literature [27,29].

Permanent capsule retention is generally due to a lesion that has given rise to the underlying symptoms and surgical intervention leads in most cases to an improvement of the clinical situation [13,19,26,28-30]. If this statement also is true for patients with CID is yet unproven. In our study no patient had a permanent retention though suffering from massive obstructive symptoms. We believe that CE can be performed in patients with CID under the same conditions as other patients with obstructive symptoms; i.e. the patient must be made fully aware that surgical intervention may become necessary in the case of finding an obstructive lesion.

## Conclusion

Capsule endoscopy can be performed in patients with suspected or confirmed chronic intestinal dysmotility. Mucosal breaks, mainly in form of erosions and in a few cases ulcerations, were found in 16 of 18 patients. The relevance of the observed mucosal abnormalities remains uncertain. We hypothesize that mucosal erosions and ulcers in CID may be due to bacterial overgrowth. Impaired motor activity was shown by a high frequency of stomach retentions, prolonged intestinal transit times, large amounts of intestinal content and frequent failure of the capsule to reach the caecum. Small bowel transit times did not significantly differ between myopathic and neuropathic CID, or to a control group in this study. Capsule endoscopy might be useful in selective cases of CID and when concomitant or a coexisting disorder in the small intestine is suspected.

## Competing interests

The author(s) declare that they have no competing interests.

## Authors' contributions

CH analysed the capsule endoscopies, collected clinical data and wrote the manuscript with contributions from GL and US.

GL was responsible for the design of the study, collected clinical data and performed the statistical analysis.

US analysed the capsule endoscopies and participated in the design and coordination of the study.

All authors read and approved the final manuscript.

## Pre-publication history

The pre-publication history for this paper can be accessed here:


